# Psychosocial determinants of functional independence among older adults: A systematic review and meta-analysis

**DOI:** 10.34172/hpp.42354

**Published:** 2024-03-14

**Authors:** Fataneh Goodarzi, Sahar Khoshravesh, Erfan Ayubi, Saeid Bashirian, Majid Barati

**Affiliations:** ^1^Department of Public Health, School of Public Health, Hamadan University of Medical Sciences, Hamadan, Iran.; ^2^Department of Nursing, Faculty of Nursing and Midwifery, Hamadan University of Medical Sciences, Hamadan, Iran.; ^3^Cancer Research Center, Hamadan University of Medical Sciences, Hamadan, Iran; ^4^Social Determinants of Health Research Center, Hamadan University of Medical Sciences, Hamadan, Iran

**Keywords:** Depression, Aged, Functional status, Self-efficacy

## Abstract

**Background:**

In current years, the increase in older population has led to creating one of the main public health challenges, worldwide. Because of the special characteristics of older adults, this age group is exposed to possible problems, such as mental and physical disorders, that usually affect their functional independence. The study aimed to determine the psychosocial determinants (e.g., depression, social support, and self-efficacy) affecting functional independence among older population.

**Methods:**

Our search was conducted on three international databases (Web of Sciences, PubMed/Medline, and Scopus) for all the observational studies (cross-sectional, cohort or longitudinal designs) on the social and psychological determinants of functional independence among older adults. Papers published in English without limitation of time were reviewed from inception to 26 August 2023. The quality assessment tool was the Newcastle-Ottawa Scale (NOS). The I2 index was used to quantify the degree of heterogeneity among the studies. In the case of heterogeneity higher than 50%, the random effects model has been used for overall estimation of the effects; otherwise, the fixed effects model was used. The pooled associations were expressed as odds ratio (OR) and 95% confidence intervals (CIs). Stata version 14 software (StataCorp LP) was used for data analysis. The significance level was considered at 0.05.

**Results:**

In the initial search, 6978 articles were retrieved, and finally, considering the inclusion criteria, 46 articles were examined. Finally, 18 articles were eligible for meta-analysis. The findings indicated that among all the determinants affecting functional independence among older adults, depression could lead to a 76% increase in functional dependence.

**Conclusion:**

The findings provide a statistically significant relationship between psychosocial factors and functional independence. Depression was the strongest determinant of functional dependence among older adults.

## Introduction

 The maintenance of the older adult’s independence in physical and mental activities is an important determinant that improves their quality of life. Therefore, a useful way of assessing health in older people is through functional assessment.^1^ Functional independence, on the other hand, is a crucial factor in determining an older person’s quality of life.^2^ Functional independence has become an important public health issue because of its impact on the quality of life of individuals, families, and health services.^3^

 Functional independence has been used to characterize a person’s capacity to carry out activities of daily living (ADLs).^4^ ADLs and instrumental activities of daily living (IADLs) are used to assess functional independence.^5^ The ADLs include eating, dressing, undressing, bathing, toileting, going to bed, waking up, and grooming. More reliable indicators of functional independence include IADLs, such as preparing food, shopping, using a telephone, housekeeping, and doing laundry, signifying functional ability among older adults, and dependence on performing each of the mentioned activities indicates a functional impairment.^6,7^

 The evidence demonstrates that the loss of independence is an important concern to older adults. Incapacitated older adults have a lower quality of life and are more prone to depression and death.^8,9^ The ability to perform daily activities is known to maintain independence and is an effective determinant in the success and older adults’ health.^1,10^ In America, Italy, and Spain, the prevalence of dependence in basic ADLs among older adults was reported to be 10%-21%, 10.6%, and 34.6% respectively.^11-13^ In addition, the prevalence of dependence in IADLs among older adults in Taiwan^14^ and Spain^13^ was 26.2% and 53.5%, respectively.

 Psychosocial determinants of ageing can have an impact on a person’s well-being, quality of life and health. The quality of life of older adults goes beyond physical health that focuses on capacity or active social participation, it can affect the performance of independent ADLs among older adults.^15^ In addition, loss of functional independence and increased incidence of chronic disease are associated with psychosocial factors such as depression, social isolation, anxiety, stress, and weak relationships.^16^According to the results of the previous studies, depression leads to a decline in performance^17-19^; nonetheless, some other studies suggest that decreased performance leads to depression. On the other side, independence among older adults is related to their mental state.^1,2,20^

 As ageing results in dramatic changes in all dimensions of the lives of older adults, a thorough understanding of these changes and their associated determinants not only improves their physical, mental and social conditions, but also plays an important role in healthy and active ageing.^21,22^ Considering that the studies on functional independence among older adults have been carried out in a specific geographical area and on a limited population in Iran and other countries, it seems necessary to conduct a systematic search and prepare a more accurate report using systematic data. This systematic review would yield more accurate results, which can significantly help experts improve functional independence and, consequently, the quality of life among older adults. Therefore, the study aimed to determine the social and psychological determinants (e.g., social support, self-efficacy, depression) affecting the functional independence (e.g., ADL, IADL) of older adults through a systematic review and meta-analysis based on observational studies.

## Materials and Methods

 This systematic review and meta-analysis adhered to PRISMA standards.^23^

###  Data sources and search strategy

 The study was conducted on three international databases (Web of Sciences, PubMed/Medline, and Scopus) for all the observational articles on the social and psychological determinants of functional independence among older people in English without limitation of time that were reviewed from inception to 26 August 2023. The search strategy was developed using Medical Subject Headings (MeSH) with the related keywords. For example, the search method used in the PubMed/Medline database was shown in Supplementary file 1.

###  Inclusion and exclusion criteria 

 The Inclusion and exclusion criteria were set based on PEO framework (Population, Exposure, and Outcome). All observational studies (cross-sectional, cohort or longitudinal designs) assessed the psychosocial determinants of functional independence among older adults. The study subjects were healthy older adults ( ≥ 60 years) without functional limitations. The exclusion criteria included the studies conducted on unhealthy people to assess the psychosocial determinants affecting functional independence, the studies that regarded functional independence as an independent variable, review articles, clinical trial studies (RCT), systematic studies, letters, interventional qualitative, and interpretive studies, case reports, meta-analyses, and studies with a final outcome other than functional independence.

###  Data extraction

**Figure 1:**
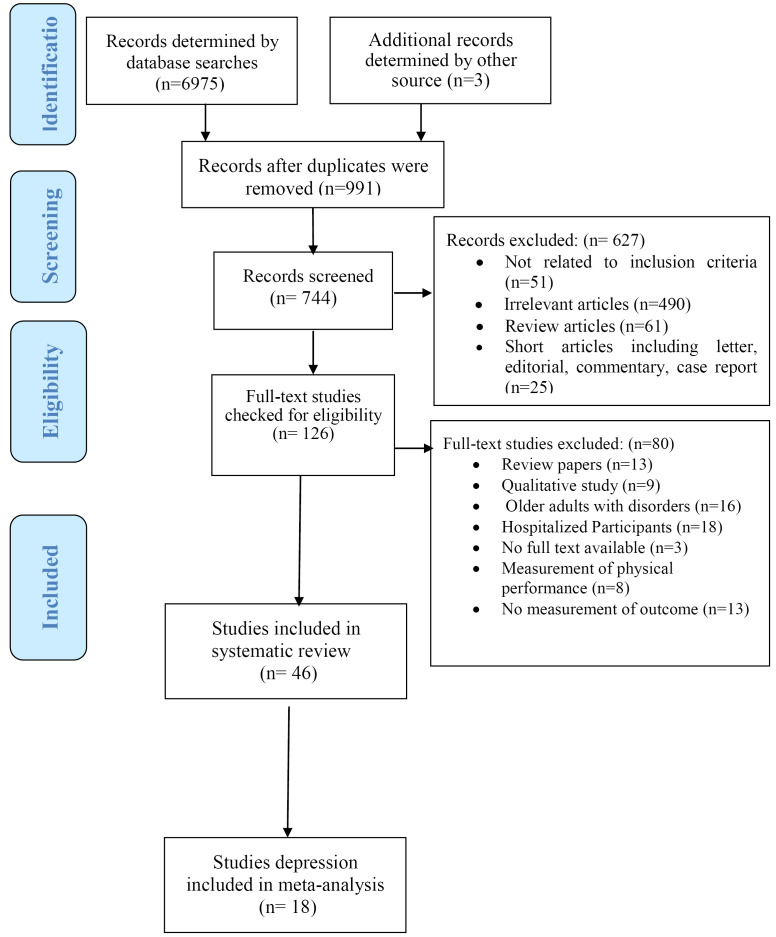
PRISMA flow diagram of the selection process for systematic reviews and meta-analysis

 The articles (titles, abstracts, and full texts) were independently searched and retrieved by two researchers. EndNote X7 (Thomson Reuters) software was used to enter the articles identified in the initial search. After eliminating duplicates, two researchers independently assessed the titles and abstracts of the obtained studies. Studies were identified based on the inclusion criteria. In case of disagreement between the researchers about the inclusion of the study, a final consensus was reached through discussion and a third person’s opinion. Then, the full text of all the studies that met the inclusion criteria was retrieved (Figure 1). The following information was extracted from the included studies: author names, year of publication, age, sex, study type and design, sample size, country of study, functional independence assessment tool, psychosocial determinants, study results, descriptive statistics (ratio/ratio, number/ratio, mean/standard deviation) and analytical statistics (a 95% confidence interval [CI], risk ratio [RR], odds ratio [OR], standard error [SE]). Then, the data of the selected articles were independently entered into the table by two researchers.

###  Quality assessment tools

 The quality evaluation tool was the Newcastle-Ottawa Scale (NOS).^24^ Two authors used this tool independently. In case of disagreement between the researchers, a final consensus was reached through discussion and a third person’s opinion. The NOS assesses the potential of bias in observational studies using three criteria: 1) study group selection (four questions), 2) group comparability (two items), and 3) exposure/outcome assessment (three items). The NOS assigns a star rating to each study, with a maximum of nine stars. An article is considered to be of high quality if it receives seven stars or more, while six stars or less indicates that the article is of low quality.

###  Statistical analysis

 The effect size indicators, such as the odds ratio, a 95% CI, and estimate standard error, were extracted from the studies that reported the adjusted effect size in terms of confounders. The I^2^ index was used to quantify the degree of heterogeneity between articles. In case of heterogeneity was greater than 50%, the random effects model was used for the overall effect estimate, otherwise the fixed effects model was used.

 A forest plot diagram was used to report the results. The funnel plot, Egger’s test, and Begg’s test were applied to determine publication bias. Subgroup analyses (study design, sample size, continent, and NOS-rating scale) of the included studies in meta-analysis were conducted. Stata version 14 software (StataCorp LP) was used for data analysis. The significance level was considered at 0.05.

## Results

###  Search results

 In total, 6975 articles were retrieved from three databases (Web of Sciences (n = 2075), PubMed (n = 568) and Scopus (4332)). Three articles were identified by the manual check by controlling references list of the included studies. A number of 991 articles were removed due to duplication. Then, the titles of the remaining 6017 articles were checked, out of which 744 articles remained for abstract review, and 627 irrelevant articles were removed. The full text of the remaining 126 studies was reviewed. After removing 80 articles, 46 articles were finally reviewed in this study. Finally, 18 studies were eligible for inclusion in the meta-analysis (Figure 1).

###  Features of the included studies 

 The included articles were as follows: 28 cross-sectional studies,^2,3,25-50^ 18 cohort studies,^19,51-67^ 26 studies in European countries,^2,19,26,27,30,33,34,41,43-47,52-56,58-61,63,64,66,68^ 18 studies in Asian countries,^3,25,28,29,31,35,37-40,42,48,50,51,57,62,65,67^ and two studies conducted in African countries.^32,36^ Moreover, the query yielded five related non-English articles, which indicated a significant relationship between psychosocial determinants and functional independence.^69-73^ Furthermore, most studies were conducted in America, Brazil, Japan, Turkey, China, and Spain, respectively. Most studies were performed after 2000, except for three studies that were conducted in 1995, 1996, and 1999.^53,55,64^

###  Results of the systematic review

 Among all the determinants affecting functional independence, depression was the most frequently measured variable and considered a reliable predictor for functional independence reduction in this population. A significant negative relationship between functional independence and depression was observed. America, Brazil, Japan, and Turkey were the countries where most studies were conducted. After depression, stress, social participation, social support, social isolation, and emotional support were among the important determinants in these studies. Most studies were carried out on older adults aged ≥ 60 years. The sample size of the studies varied from 27 to 5050 cases. All studies, except the one by Almeida et al,^63^ were conducted on both genders.

 Moreover, 48% of the studies used the Katz or Lawton tests or both.^2,3,19,25,27,29,30,33,37-39,45,51-54,57,64-68^ Therefore, Katz and Lawton scale was the most frequently used questionnaire for measuring functional independence in these studies. The geriatric depression scale (GDS) was another widely used questionnaire in the studies. Furthermore, 22% of studies referred to other psychological determinants, such as emotional status, mental health, affect, self-efficacy, cognitive status, anxiety, and psychological distress as the best predictors of decreasing functional independence in older adults.^2,26,27,34,40,41,44,51,53,60^

 About 33% of studies investigated the social determinants related to functional independence, including social support, social participation, self-care, social isolation, and social capital.

 A significant relationship between these social determinants and functional independence was found in the studies. Social determinants were more frequently investigated in America and China than in other countries.^2,3,25,26,29,35,37,38,51,53,54,57,60,65,66^

Supplementary file 2 provides details of the studies included in this systematic review.

###  Meta-analysis results 

**Figure 2:**
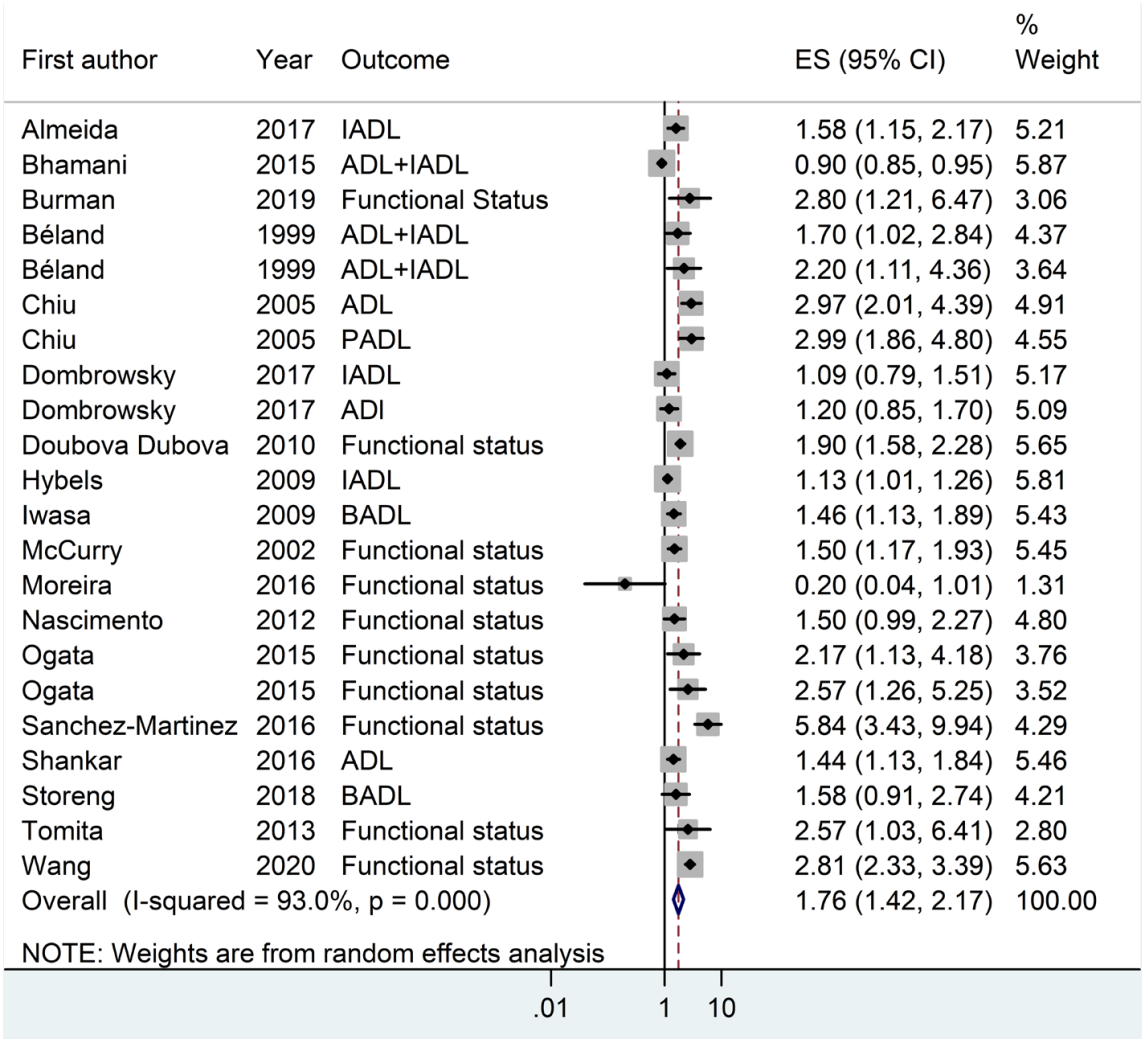
Forest plot for meta-analysis of association between depression and Functional independence

**Figure 3:**
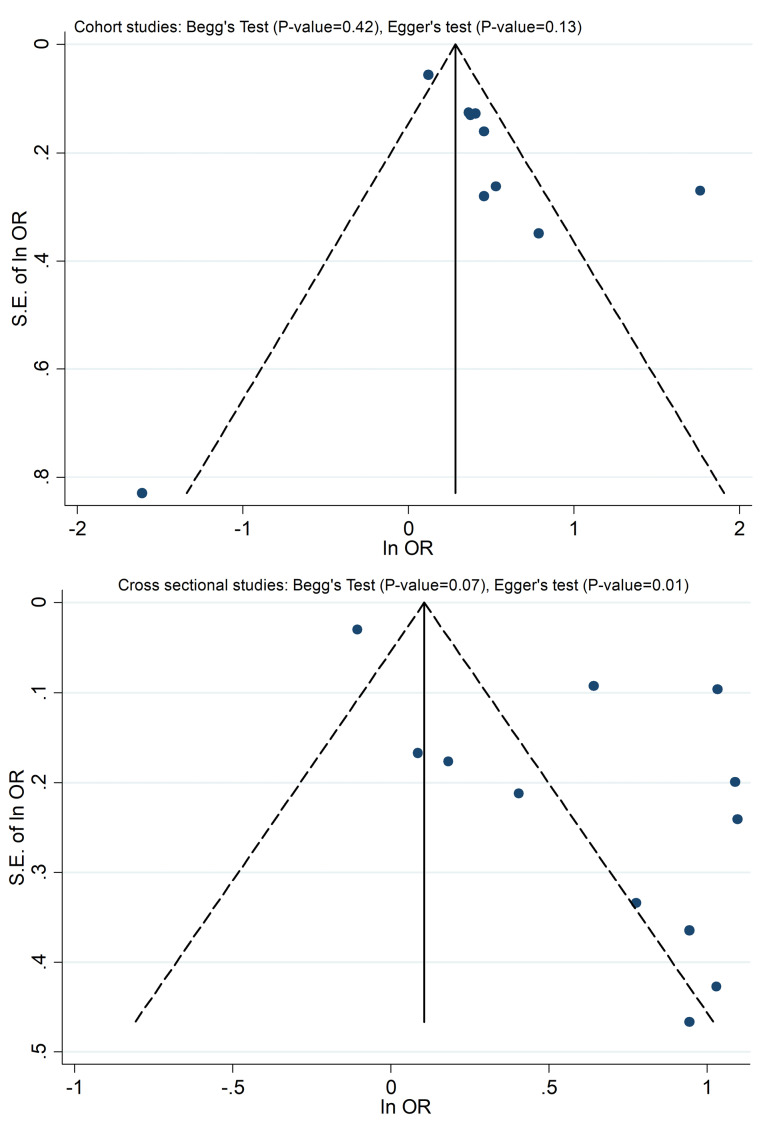
Funnel plot for assessing the risk of publication bias of studies included in the meta-analyses: Relationship between depression and functional independence among older adults

**Figure 4:**
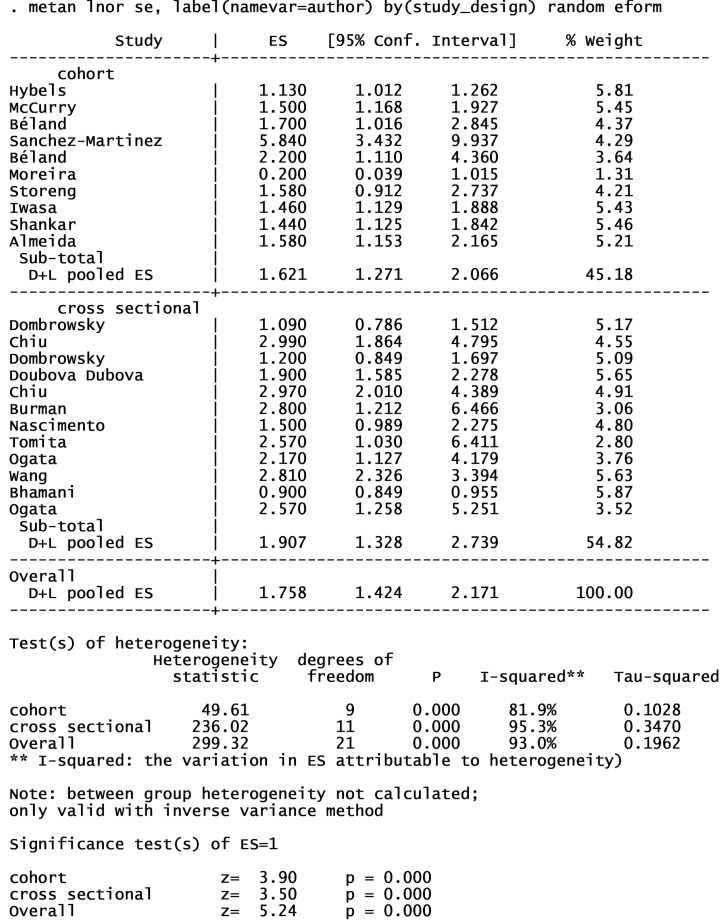
Subgroup analysis according to study design

**Figure 5:**
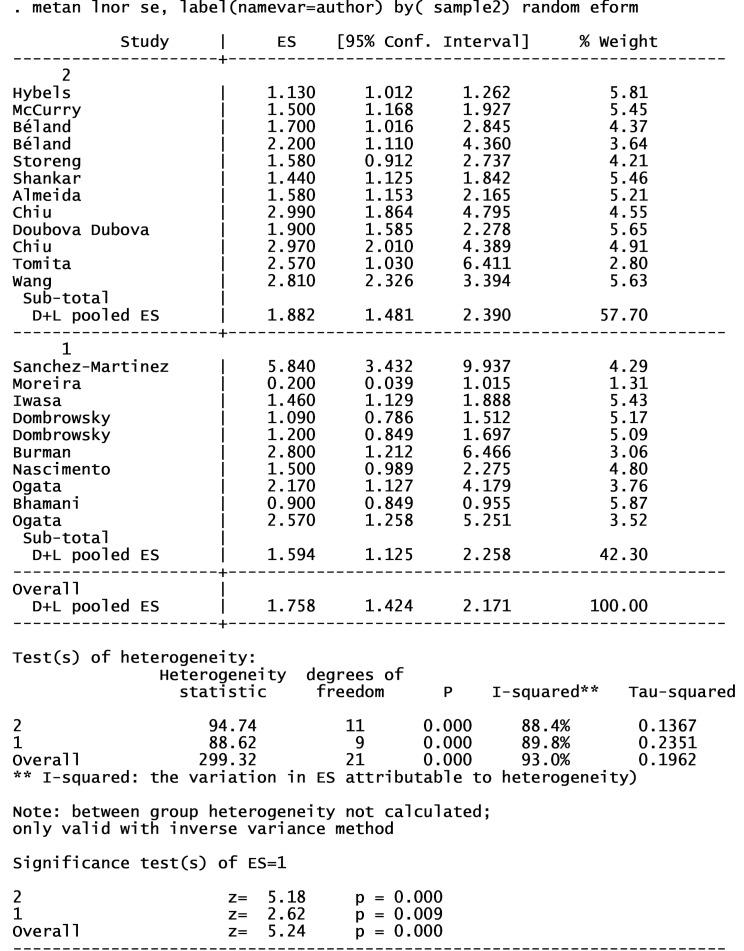
Subgroup analysis according to sample size

**Figure 6:**
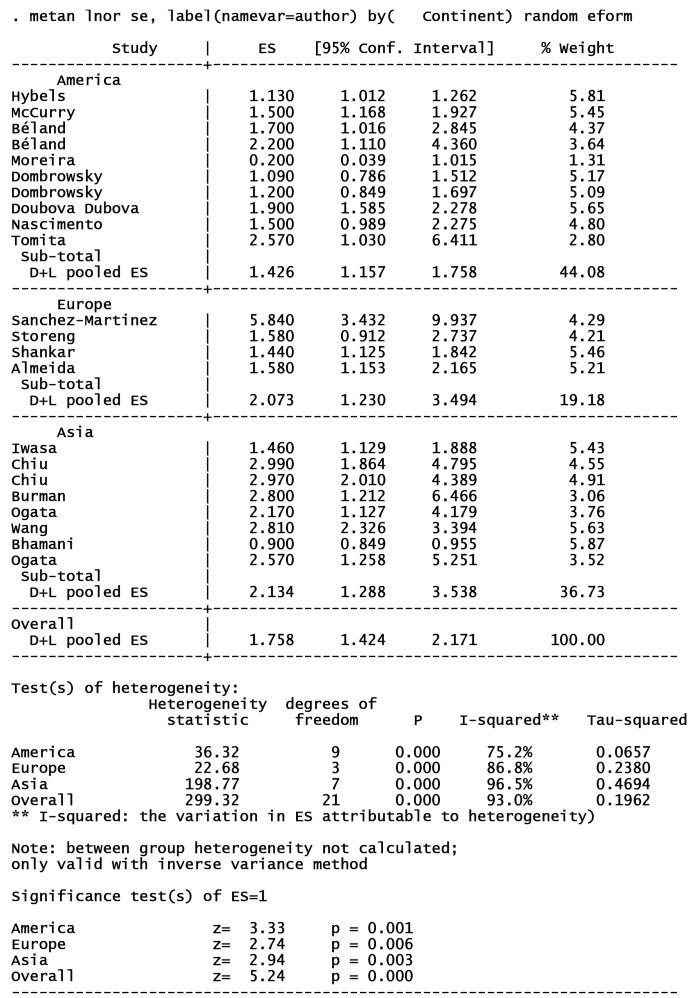
Subgroup analysis according to continent

**Figure 7:**
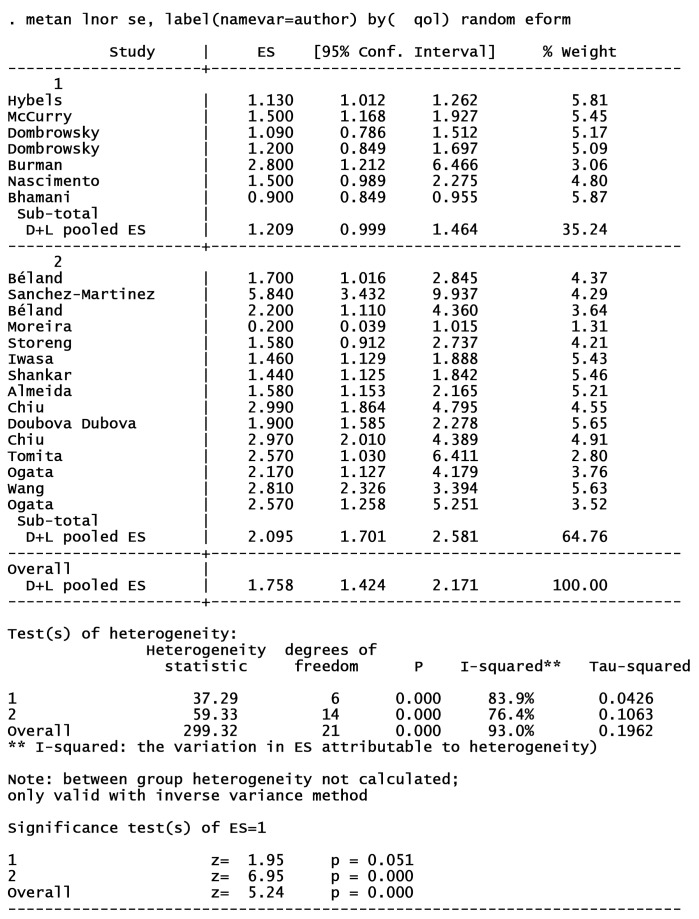
Subgroup analysis according to NOS-rating scale

 18 articles from 46 studies reported an adjusted effect size index for the association between depression and functional independence and were included in the meta-analysis (Figure 2).^2,3,19,30-32,39,42,49,52,54,59-64,67^ The results pointed out that depression can increase the risk of functional dependence by 76% (I^2^ index: 93% with a significance level of less than 0.001). There was no publication bias in cohort studies; however, some degrees of publication bias were observed in cross-sectional studies (Figure 3). The results of subgroup analyses of are shown in Table 1. Subgroup analyses based on study design, sample size, continent, and NOS-rating scale are presented in Figures 4, 5, 6 and 7 respectively.

**Table 1:** Subgroup analyses of included studies in meta analyses

**Variables **	**No of studies**	**RR (95% CI)**	**Weight (%)**	**I** ^ 2 ^ ** %**	***P*** ** for heterogeneity**
Study design					
Cohort	9	1.62 (1.27, 2.06)	45.18	81.9	< 0.001
Cross sectional	9	1.90 (1.32, 2.74)	54.82	95.3	< 0.001
Sample size					
< 1000	8	1.59 (1.12, 2.25)	42.30	89.8	< 0.001
> 1000	10	1.88 (1.48, 2.39)	57.70	88.4	< 0.001
Continent					
Asia	6	2.13 (1.28, 3.53)	36.73	96.5	< 0.001
America	8	1.42 (1.15, 1.75)	44.08	75.2	< 0.001
Europe	4	2.07 (1.23, 3.49)	19.18	86.8	< 0.001
NOS-rating scale					
< 6	6	1.20 (0.99, 1.46)	35.24	83.9	< 0.001
≥ 6	12	2.09 (1.70, 2.58)	64.76	76.4	< 0.001

## Discussion

 This study aimed to determine the psychosocial predictors (e.g., social support, self-efficacy, and, depression) affecting the functional independence (e.g., ADL, IADL) of older adults. In this review, 46 studies were reviewed. Totally, the results were categorized in two sections of psychological and social determinants. Psychological determinants were depression, emotional status, mental health, affect, self-efficacy, cognitive status, anxiety, and psychological distress. Social determinants included social support, social participation, self-care, social isolation, and social capital.

 Among psychological determinants, depression was the most frequently investigated determinant. Evidence indicates that depression is one of the psychological conditions related to functional independence among older adult. As the psychological status of older adults is closely related to functional independence.^74^ Therefore, depression is one of the most important predictors of functional independence in older adult population.^56^ According to the previous study, the most important feature of depression is mood swings, which can lead to disability in performing ADLs among older adults.^20^ Furthermore, Boga and Saltan reported that the levels of dependence on activities in daily living were related to mental status and depression among older adults. In addition to the financial cost, depression in older adults has negative consequences.^17,40^ Also, depression reduces the quality of life in older adults, and lower quality of life is associated with more severe ADL and IADL problems.^75^ Therefore, depression, as one of the psychological determinants affecting functional independence, should be prioritized in preventive activities.^1^ This is because depression in older people can be reduced by performing ADLs.^20^ According to Storeng et al, the most significant risk variables for needing assistance with one or more basic/instrumental daily living activities were life satisfaction, depression, and health perception. These elements can be among the potential targets for preventative measures.^61^ The fact that America places more emphasis on the issue of depression than other countries may be because it recognizes the problem of depression in older adults as a serious public health issue due to the rapidly growing older population in the country and the resulting increase in demand for mental health services.^76^ According to studies, older adults’ ability to perform daily living activities is a good indicator of their likelihood of experiencing depression. As a result, by carefully assessing their health and functional ability, older adults’ depression and physical inability to perform daily living activities can be avoided.^1,56^ On the other hand, it seems that there is a major difference between countries, both developed and developing in the readiness of their government and people to face the challenge caused by the increasing number of older adults and developed countries are more prepared in this regard. Nonetheless, most developing countries lack the sufficient and necessary knowledge about this issue, additionally, its consequent health, economic and societal issues.^77,78^

 The findings of our research showed that other effective psychological determinants of functional independence were emotional status, mental health, affect, self-efficacy, and psychological distress, which play an effective role in functional independence. These determinants are strong predictors for functional independence. Intervention strategies based on these determinants have been shown to successfully improve functional independence among older adults. It’s crucial to consider certain elements in older people at risk for declining functional independence. Therefore, it is proposed to include them in programs aimed at preventing the decreased of functional independence among older adults. Another effective determinant in this regard was self-efficacy, which alludes to a person’s belief in his/ her capacity to perform desired activities.^79^ In general, the studies indicated that self-efficacy is significantly correlated with functional status in older adults, suggesting that self-efficacy is especially important for maintaining functional independence among older adults. So, self-efficacy can be expanded as an intervention target in all older people who are widely potentially functionally declining; moreover, some programs should be developed to prevent functional decline. Accordingly, those who are more confident in their abilities and have higher self-efficacy will exhibit better performance.^35,40,48,51,53,70,80^ Also, considering the growing trend of aging in the world, it is important to pay attention to psychosocial determinants. Self-efficacy not only increases social participation among older adults but also affects the improvement of their quality of life.^79,81^ Mendes de Leon et al addressed that self-efficacy has a positive effect on functional independence, in such a way that high self-efficacy is especially indicative of older people’s functional independence.^53^

 The findings of this investigation also revealed that social determinants have a positive and important connection with functional independence among older adults. This means that high levels of these determinants promote functional independence among older people. In fact, one of the important social determinants that influence older individual’s ability to function independently is social support. Social support is among the social determinants of health, which has received particular attention in recent years.^79^ Social support can be effective in the mental judgment about stressful determinants, feeling safe and tranquil, self-esteem, as well as increasing personal and social skills among older adults. It is described as a network of communication that promotes health behaviors.^82^ It is therefore considered an important determinant in preserving bodily and mental health and participation in life.^83,84^ The older adults who are in contact with others and use more information sources to solve daily problems have more independence in their daily activities.^83^ Actually, the level of health increases with improvement in social support. Therefore, health is closely linked to social support, which is regarded as a predictor of the connection between daily activities and depression.^84^ As a result, there is a immediate and important relationship between social support and performance daily activities among older adults. That is to say, an increase in social support results in higher levels of engagement in daily activities.^85,86^ Hajek concluded to enhance social support may help seniors preserve their functional abilities.^66^

 In our study, 48% of the studies used the Katz and Lawton measurement tool. This instrument was designed in 1969 to measure physical performance among older adults using a dual rating (dependent/independent) and is known as a standard tool to measure the performance of older people in the form of a full spectrum of abilities in different societies.^87^ Moreover, this tool can predict well the decline of being able to perform ADLs and recovery after that due to older adults. The present study’s findings also demonstrated that this questionnaire can be used by researchers as a useful and trustworthy tool in most countries.

 The goal of the current meta-analysis study was to examine the connection between older persons’ functional independence and depression. The outcomes showed that depression can lead to a 76% increase in functional dependence. The most prevalent and significant factor detrimentally affecting psychosocial functioning in older persons might be considered to be depression, a condition characterized by feelings of sadness and hopelessness. Depression in older persons is linked to lower functional levels, a worsening of health, and a lower quality of life.^88,89^ The relationship between daily activity and psychological determinants, including depression, has been highlighted in multiple studies. According to certain research, older persons who were unable to do at least one essential daily task had a lengthy history of depression.^90-92^ Therefore, decision-makers in the field of health can benefit from having a complete grasp of older persons’ psychosocial state and how it affects their functional independence in order to plan and implement effective interventions to maintain and raise the level of daily activities in older individuals.

## Strengths and weaknesses

 This study is the first systematic review to particularly look at the psychosocial determinants of functional independence among older persons is the current study’s most significant strength. Most of the reviewed studies had assessed the impact of a limited how many psychological or social determinants on functional independence; nonetheless, the present research provided strong evidence of the most important psychosocial determinants of functional independence among older people in a relatively comprehensive systematic evaluation.

 Therefore, the results of this study can be useful for researchers in implementing intervention programs to promote independence in older people. Among the notable limitations of this study, we can refer to the mere inclusion of articles published in the English language. Second, our study is not exempt from risk of bias. One of the reasons may be non-English studies being excluded from our studies. Also, most of the included studies had cross-sectional design. This can have an effect on the study results. The third limitation was the lack of access to the full text of some articles. Forth, there could be some selection bias in the results that overlooks unpublished studies, gray texts, studies found in other databases, and perhaps even non-English papers.

## Conclusion

 The results of this study found a statistically significant relationship between psychosocial determinants and functional independence among older adults. Since functional independence among older adults is a multi-dimensional concept that is affected by various determinants, the identification of these determinants can help design and implement interventions related to the prevention, control, and management of determinants that lead to dependence in older adults. Additionally, depression has a considerable effect and is a reliable indicator of the decline in functional independence in older persons when compared to other psychosocial variables. Consequently, depression and physical disability to perform ADLs in older adults can be prevented by a careful examination of their health and functional capacity for medical teams and their household members. So, depression and functional independence to perform daily living activities among older adults can be prevented by a careful examination of their functional capability by health providers and their family.

## Acknowledgements

 The Vice-Chancellor for Research and Technology at Hamadan University of Medical Sciences is thanked by the authors for his cooperation and provision of the facilities required to carry out this work.

## Competing Interests

 The authors claim to have no conflicts of interest.

## Ethical Approval

 The current study was approved by the Research Ethics Committee of Hamadan University of Medical Sciences (No. IR.UMSHA.REC.1400.980).

## Supplementary Files


Supplementary file 1. Search strategy in PubMed.

Supplementary file 2. Summary of included studies
