# Supplementary material for: Psychosocial determinants of functional independence among older adults: A systematic review and meta-analysis
Source: Health Promot Perspect. 2024 Mar 14;14(1):32–43. doi: 10.34172/hpp.42354 (PMC11016145; doi:10.34172/hpp.42354)
Supplement: Supplementary file 1 — Search strategy in PubMed. [file hpp-14-32-s001.pdf]

Goodarzi et al, **Health Promotion Perspectives,** 2024, 14(1), 32-S1
doi: 10.34172/hpp.42354
https://hpp.tbzmed.ac.ir

Supplementary file 1

Search strategy in the database PubMed

**Psychological factors**	(TITLE-ABS-KEY (depression) OR TITLE-ABS-KEY ("Depressive disorders") OR TITLE-ABS-KEY ("Major depressive disorder") OR TITLE-ABS-KEY (attitude) OR TITLE-ABS-KEY ("Self Efficacy") OR TITLE-ABS-KEY (knowledge) OR TITLE-ABS-KEY (intention) OR TITLE-ABS-KEY ("Subjective norm") OR TITLE-ABS-KEY (belief) OR TITLE-ABS-KEY (stress) OR TITLE-ABS-KEY ("self-esteem")
**social factors**	TITLE-ABS-KEY ("social support") OR TITLE-ABS-KEY ("Social Networking") OR TITLE-ABS-KEY ("Social Capital") OR TITLE-ABS-KEY ("Social Isolation") OR TITLE-ABS-KEY ("Self Care")
**Functional independence**	TITLE-ABS-KEY ("Functional Status") OR TITLE-ABS-KEY ("functional dependency") OR TITLE-ABS-KEY ("Functional Independence")
**Older adults**	TITLE-ABS-KEY (elderly) OR TITLE-ABS-KEY ("older age") OR TITLE-ABS-KEY ("older adults"))

ABS: Abstracts, KEY: Keywords, OR: Boolean operators
