# Supplementary material for: Psychosocial determinants of functional independence among older adults: A systematic review and meta-analysis
Source: Health Promot Perspect. 2024 Mar 14;14(1):32–43. doi: 10.34172/hpp.42354 (PMC11016145; doi:10.34172/hpp.42354)
Supplement: Supplementary file 2 — Summary of included studies [file hpp-14-32-s002.pdf]

Goodarzi et al, **Health Promotion Perspectives,** 2024, 14(1), 32-S2
doi: 10.34172/hpp.42354
https://hpp.tbzmed.ac.ir

Table 1. Summary of included studies

First author (year of publication-country)	**Age**	**Gender**	**Study design**	**Sample size**	**Determinants**		**Outcome**	**Descriptive measures**	**Analytic measures/conclusion**
					**Psychological**	**Social**			
Dombrowsky et al. (2017- USA) [2]	>65	men: 24(26%)<br><br>women: 68(74%)	cross-sectional	92	cognitive status Geriatric Depression Scale–Short Form (GDS-SF),	Engagement with Meaningful Activity Survey (EMAS),<br>social support	Functional Comorbidity Index (FCI)<br>Katz Index of Independence in ADLs (Katz)<br>Lawton–Brody IADL Scale (Lawton–Brody).	Mean (SD), n<br>FCI: 4.07 (2.23), n = 92<br>Katz: 5.67 (0.56), n = 91<br>Lawton–Brody:<br>7.47 (1.06), n = 92	OR (95% CI)<br>Lawton–Brody model<br>FCI score: 0.889 (0.681–1.147) EMAS score: 1.183 (1.037–1.376)<br>GDS score: 1.090 (0.794–1.521)<br>Katz model<br>EMAS score: 0.922 (0.814–1.035)<br>FCI score: 0.837 (0.664–1.040)<br>GDS score: 1.204 (0.883–1.766)
Burman et al (2019- India) [3]	>60	men: 142(57.7)<br>women: 104(42.2)	cross-sectional	246	depression	Marital status, Family type	Functional Status (ADL and IADL)	32.4% and 59.3% were dependent for basic ADL and IADL respectively	**AOR (95% CI)**<br>Widowed/separated<br>2.3 (1.1-5.2)<br>joint family<br>2.6(1.1-5.9)<br>depressed<br>2.8(1.2-6.4)<br>had increased odds of dependency for ADL and IADL
Koc et al (2015- Turkey) [25]		Female: 208 (55.9)<br>Male: 164 (44.1)	Cross sectional	372	-	Social assurance (research made question)	ADL and self-care agency (ESCA)	ADL: n (%)<br>Dependent<br>54 (14.6)<br>Semidependent<br>69 (18.5)<br>Independent<br>249 (66.9)	There was a negative correlation between Social assurance and self-care agency:<br>Adjusted regression coefficient=-0.446
Martin et al (2013- USA) [26]	98	Male: 41(17.5)<br>Female: 193(82.5)	Cross sectional	234	Affect (Bradburn Affect Balance Scale)	Conscientiousness (NEO),	Functional capacity (OARS)	Functional Capacity:<br>Mean (SD):<br>14.71 (7.50)	Adjusted regression coefficient (SE)<br>Positive affect: 0.46 (0.14)<br>Conscientiousness: 0.01 (0.03)<br>Social Provisions: 0.03 (0.12)

First author (year of publication-country)	Age	Gender	Study design	Sample size	Determinants		Outcome	Descriptive measures	Analytic measures/conclusion
					Psychological	Social			
						Social Provisions (Social Provisions Scale)			
Mattos et al (2014-Brazil) [27]	76.6 ±9.4	Male: 460 (60.5) Female: 539 (70.9)	Cross sectional	760	Cognition (MMSE) Emotional aspects		Functional capacity (ADL and IADL)	Prevalence of dependence ADL: 382 (50.3%)<br><br>IADL: 617 (81.2%)	**Adjusted OR (95% CI)**<br>**ADL as outcome**<br>cognitive aspects (Temporal orientation; 4 and 5 as reference)<br>0 and 1: 4.41 (1.46 - 13.38)<br>2 and 3: 1.21 (0.38 – 3.82)<br>cognitive aspects (reading no vs. yes): 0.22 (0.07-0.63)<br>emotional aspects ((Have you been upset lately? No vs. yes): 0.21(0.06 – 0.70)<br>**IADL as outcome**<br>Emotional aspects (Do you often feel abandoned? Yes vs. no)<br>1.82 (1.01 – 3.27)
Morala et al (2006-Japan) [28]	65 - 80	Male: 77(38.5) Female: 123(61.5)	cross-sectional	200	Depression (GDS)		functional status (PPT)	Physical Performance Test<br>N (%)<br>200 (100%)<br>Mean ±SD<br>19.3 ± 3.9<br>(95% CI)<br>18.7 – 19.8<br>The PPT mean score of this population was 19.3 (SD= 3.9)	GDS (β = -0.20, p = 0.004) was significantly associated with functional level.<br>GDS had a negative significant correlation with PPT (r= - 0.16, p<0.05)
Murat et al (2019-Malaysia) [29]	60-88	Male: 123 (47.7) Female: 135 (52.3)	cross-sectional study	258		Social relations and Social participation (Two self-reported items)	functional status (IADL)	The prevalence of IADL disability 58.1%.<br>mean±SD IADL ability 6.76±1.37	Adjusted OR (95% CI)<br>Visiting friends and/or relatives (No vs. Yes)<br>5.7 (0.7, 48.0)<br>Taking part in activity/social programs (No vs. Yes)<br>1.8 (0.3, 5.8)
Nascimento et al (2012-Brazil) [30]	> 60	male: 290 (46.7) Female: 331 (53.3)	cross-sectional	621	history of depression		functional ability (ADLs and IADLs)	The prevalence functional ability<br>16.2%<br>(95% CI)<br>(13-19%)	**Multivariate model; Prevalence Ratios (95% CI)**<br>history of depression<br>1.5 (1.0–2.3)

First author (year of publication-country)	Age	Gender	Study design	Sample size	Determinants		Outcome	Descriptive measures	Analytic measures/conclusion
					Psychological	Social			
Ogata et al (2015-Japan) [31]	≥65	Men : 233 (42.8) Women 312 (57.2)	cross-sectional study	545	Depressive state		Functional Capacity	TMIG M (SD) for men: 7.98 (3.67) for women: 9.16 (3.67)	Depressive state was significantly associated with impaired higher-level functional capacity in both men and women. The marginal odds ratios were OR (95% CI) for men 2.17 1.13–4.19) for women 2.57 (1.26–5.26)
Tomita et al (2013-African) [32]	>65	Male: 479(38.0) Female: 950(62.0)	Cross sectional	1,429	Depression		Functional status (ADL, IADL, and PFM)	ADL Dependency 59(3.6%), IADL Dependency 346 (21.4%), PFM Dependency 635 (39.3%).	functional dependence in ADL adjusted OR (95% CI) 2.57 (1.03-6.41) IADL 2.76 (1.89-4.04), PFM 1.66 (1.18-2.33) but the relationship between depression and functional status, particularly PFM, appeared weaker in older age
Uchoa et al (2019-Brazil) [33]	>60	Female: 74 Male: 26	cross-sectional	100	Depression		functional capacity (IADL)	Functional dependency of older adults for performing IADL was estimated at 46%.	a moderate negative correlation was found between the results obtained with the Geriatric Depression Scale (EDG-15) scale and the Lawton and Brody Scale (R^2= -0.4121 $p < 0.05$)
Van der Weele et al (2009- Netherlands) [34]	90	men: 56(27.8) women: 145(72.1)	Cross sectional	201	Depression anxiety		functional status ADL IADL	Disabilities in ADLs (GARS): 36 (17.9%)	Disabilities in ADLs (GARS) depending on the presence of depression: (median, Interquartile range) Depressed subjects and anxiety present: 44 (34–53) Depressed subjects and anxiety absent: 53 (40–60) p-value=0.10 Non-depressed subjects and anxiety present: 34 (30–52) Depressed subjects and anxiety absent: 34 (27–42) p-value=0.25
Wang H et al. (China-2013) [35]	> 60	Male: 719 (46.6) Female:823 (53.3)	Cross-sectional	1542		living arrangements social support emotional support	functional disability (BADL, IADL, and ADL)		unmarried living alone, ß for BADL: -1.262, ß for IADL: -2.112, ß for ADL: -3.388; for all , P< 0.001 Compared with the married living with children only,

First author (year of publication-country)	Age	Gender	Study design	Sample size	Determinants		Outcome	Descriptive measures	Analytic measures/conclusion
					**Psychological**	**Social**			
									ß for BADL: -1.166, P<0.01<br>ß for IADL: -2.723, P< 0.001<br>ß for ADL: -3.902, P< 0.001<br>Emotional support<br>(some difficulty vs. severe difficulty)<br>ß for BADL: -1.94<br>ß for IADL: -2.02<br>ß for ADL: -3.22<br>for all P< 0.001<br>No difficulty vs. severe difficulty<br>ß for BADL: -1.325<br>ß for IADL: -2.300<br>ß for ADL: -3.635<br>for all, P< 0.001
Akosile et al (2018- Nigeria) [36]	60-98	men: 90(43.7) women: 116 (56.3)	Cross-Sectional	206	Depression		Functional Disability (ADL) Functional status Questionnaire	45.5% depression.<br>at least 30% had functional disability in at least one domain	Depression had significant but inverse correlation with FSQ (r= -0.542 - -0.705; p<0.001) domains.
Asim et al (2021- Pakistan) [37]	> 60	men: 214(50.60) women: 209 (49.40)	Cross-sectional	423		Living with family	functional status (IADL, LADL, TD)	Independent in doing their all-routine work: 42.8%<br>The physical functional status decline<br>Partially<br>35.2%,<br>completely 22.0%	
Bai et al (2020- China) [38]	> 60	men: 770(42.5) women: 1040(57.4)	cross-sectional	1810	-	Social Capital<br>Social participation, social connection social support	functional ability (ADL/IADL)	43% of whom had functional disability	**Adjusted OR (95% CI)**<br>lower social participation<br>1.60(1.26–2.03)<br>lower social connection<br>1.74(1.34–2.25)<br>Social support<br>0.73(0.57–0.94)
Bhamani et al (2015- Pakistan) [39]	≥ 60	men: 506(53.2) women: 447(47.05)	cross-sectional	950	Depression		functional status	Mean ADL score (9.9 ± 0.2 vs. 9.6 ± 0.2) was higher in men than women.	A one unit increase in ADL score showed a 10% decrease in depression after adjusting for other variables<br>**Adjusted OR (95% CI)**<br>0.9 (0.8-0.9)

First author (year of publication-country)	Age	Gender	Study design	Sample size	Determinants		Outcome	Descriptive measures	Analytic measures/conclusion
					Psychological	Social			
Boga and Saltan (2020-Turkey) [40]	65- ≥75	Men 84 (45.4%) women 101 (54.6%)	cross-sectional	185	Sleep depression mental status	-	ADL		The dependency levels in daily life activities were found to be associated with mental status (r=0.317, p=0.001) and depression (r= -0.297, p=0.003) in older adults living in nursing homes, while it was associated with mental status (r=0.439, p=0.000), sleep (r= -0.349, p=0.001) and depression (r= -0.407, p=0.000) in those living in home.
Cahn-Weiner et al (2000- Rhode Island) [41]	mean age of 81.9 (± 5.5)	men 5(18.5) women 22(81.4)	cross-sectional	27	Depression severity Emotional Status	-	Functional Status IADLs	Based on a median split of the The IADLs Performance Tasks of the Occupational Therapy Assessment of Performance and Support (OTAPS) score, subjects were assigned to a high IADL performance group (mean = 81.7) and a low IADL performance group (mean =73.9)	A total of 49% (Adjusted R2 = .37) of the variance in OTAPS score was accounted for in the final model, and the Executive Composite and GDS–S score were the only significant predictors.
Chiu et al (2005-) Taiwan) [42]	> 65	men 529(52.6) women 476 (47.3)	cross-sectional	1005	Depressive symptoms	-	functional status	Urben:<br>n %<br>(Limitation in ADL: 67 (9.9)<br>Limitation in IADL: 248 (36.6)<br>Rural:<br>[(Limitation in ADL: 34 (10.4)<br>Limitation in IADL: 100 (30.6)	Functioning disability<br>PADL<br>**Total:** 2.99 (1.87, 4.81)<br>**Urban:** 3.10 (1.76–5.49)<br>**Rural:** 3.71 (1.41–9.76)<br>ADL<br>**Total:** 2.97 (2.01, 4.39) times,<br>**Urban:** 2.81 (1.78–4.44)<br>**Rural:** 3.12 (1.34–7.30)
Fillenbaum et al (2010-USA) [43]	≥60	men 2366(33.6) women 4592(65.2)	cross-sectional	7040	Depression	Social ties (Living arrangement : Live with others)	Functional Status ADLs	Mean number of ADL problems was 0.67 (1.08). Nearly 40% needed help with one or more ADLs	**For Depression**<br>**OR (99% CI)**<br>Household tasks: 1.80 (1.54, 2.09)<br>Medication: 2.54 (2.00, 3.21)<br>Mobility: 2.11 (1.64, 2.70),<br>Personal Hygiene: 2.26 (1.69, 3.02)<br>Feeding self: 2.32(1.46, 3.68)<br>**For Living arrangement:** Household tasks

First author (year of publication-country)	Age	Gender	Study design	Sample size	Determinants		Outcome	Descriptive measures	Analytic measures/conclusion
					Psychological	Social			
									1.52 (1.23, 1.89)<br>Medication : 2.41 (1.67, 3.47)<br>Mobility: 1.75 (1.22, 2.51)<br>Personal Hygiene : 2.57 (1.63, 4.05)
Simone et al (2013-USA) [44]	Mean (*SD*) 74 (10.5) range : 51–102	Participants were predominantly female (82%)	cross-sectional	95	Affect	Frailty Status Social, Leisure Engagement, Solitary Leisure Engagement	Functional Status	-	Groningen Frailty Indicator (standardized canonical correlation function coefficient Coef=0.759 structure coefficient rs=0.844, squared structure coefficient r^2_s=0.712)<br>GFI Leisure Engagement (Coef= 0.522, r= -0.663, r^2=0.440),<br>GFI Solitary Leisure Engagement (Coef=0.051, r=-0.274, r^2=0.075).
Suchy et al (2011-USA) [45]	60 to 87	62% female 38% male	cross-sectional	75	Depression		IADLs	35% of the sample reported having one or more difficulties with IADLs. Similarly, 35% of the sample made one or more errors on performance based IADL assessment	The correlations were significant between GDS total with<br>IADL (r=0.385 p<0.001)<br>TIADL performance<br>(r=0.359<br>p<0.001)
Vankova et al (2008-Czech Republic) [46]	60	-	Baseline data Of randomized controlled trials(Cross-sectional)	308	Depressive symptoms		Functional status	Barthel Index, mean (SD): 89.22 (15.14)	The correlation between GDS 15 and Barthel Index was significant (r= -0.28, p<0.001)
Wong et al (2019-USA) [47]	57–85	-	cross-sectional	953		Social Engagement Social Relationships	Functional Status IADL/ADL	35% of wives and 29% of husbands report difficulties with IADLs or ADLs.<br>Functional status (%)<br>**(Wives, Husbands)**<br>No limitation (64.7, 71.2)<br>Any IADL, but no ADL<br><br>(20.8, 16.5)<br><br>Any ADL (14.5, 12.3)	Couples' functional limitations are associated with marital, family relationship, and friendship quality

First author (year of publication-country)	Age	Gender	Study design	Sample size	Determinants		Outcome	Descriptive measures	Analytic measures/conclusion
					**Psychological**	**Social**			
Harigane et al (2017-Japan) [48]	>65	men: 8141(47.6)<br>women: 8951(52.3)	cross-sectional	17,092	psychological distress	-	functional independence	Functional independence:<br>High: 16 149 (94.5%)<br>Low: 943 (5.5%)<br>p<.001	AOR (95% CI)<br>2.32(1.97, 2.73)
Doubova Dubova et al (2010- México) [68]	≥ 60	men: 1587(47.4)<br>women: 1761(52.5)	cross-sectional	3348	depression	Social networks types	functional dependency (BADL and/or IADL)	Dependent: 475 (14.2 %)	Older adults with functional dependency more likely belonged to a widowed network<br>(adjusted prevalence ratio 1.5; (95% CI) (1.1-2.1)<br>Multivariate<br>Adjusted Prevalence Ratio (95% CI)<br>Probable depression<br>1.9 (1.6 - 2.3)<br>Established depression<br>2.7(2.1 - 3.3)
Şahin Onat et al (2014-Turkey) [50]	> 65	Female: 93(58)<br>Males: 67(42)	cross-sectional	160	depression		functional status	-	Correlation Between Depression Scores and FAS was significant (r= -0.732, p=0.001)<br>**Linear Regression Analysis**<br>**FAS**<br>**Beta**= -0.503<br>**r=** 0.783<br>**%95**= -0.566<br>**CI**= -0.440<br>**f=**<br>(1.157) 248.22<br>**p**<0.001
Hybels et al (2009-USA) [19]	>65	men: 1017 (33.3%)<br>women: 2035	longitudinal study	4162 Base line<br>3052 first interval	Depressive Symptoms	Perceived social support	Functional Decline	Mean (SD) ADL Limitations at BL: 0.15 (0.61)<br>IADL Limitations at BL: 0.50 (1.11),<br>Mobility Limitations at BL: 0.79 (1.07).	Having 6+ depressive symptoms predicted an increase of 0.12 IADL limitations 3–4 years later (p=0.03)
Kondo et al (2007-Japan) [51]	76.46.94	Female: 280 (48%)<br>Male: 301 (52%)	Baseline data of a cohort study	581	Mental health (Mental Health component of the Medical Outcomes	Engagement status (engagement in the Mujin)	Functional Capacity (TMIG-IC)	-	**Adjusted** OR (95% CI)<br>**TMIG-IC was outcome**<br>Mujin engagement score (Non participation as reference)<br>Low: 1.01 (0.58, 1.77)<br>Middle: 1.03(0.57, 1.86)

First author (year of publication-country)	Age	Gender	Study design	Sample size	Determinants		Outcome	Descriptive measures	Analytic measures/conclusion
					**Psychological**	**Social**			
					Study Short Form-36 Health Survey questionnaire)				High: 1.73(0.99, 3.02)<br>**Mental health**<br>No risk 1.00 (reference)<br>Mild: 0.75 (0.36, 1.57)<br>Moderate: 0.67(0.33, 1.37)<br>Severe: 0.35(0.18, 0.69)
McCurry et al (2002-USA) [52]	65	Men: 966(46)<br>Women: 1128(53)	Cohort	2,094	Depression (CESD)		Functional Decline<br>Katz ADL-Branch IADL and decline in physical functioning Rosow-Breslau and Nagi scales	No decline in physical function at 2-year follow-up (81%) and 4-year follow up (75%)<br>no decline in ADL/IADL status at 2-year follow-up (94%) and 4-year follow up (89%)	**Adjusted OR (99% CI)**<br>decline in physical functioning as outcome<br>Depression: 1.19 (1.05, 1.93)<br>Major Limitation in Physical Function (five or more functional limitations) at 4-year follow up as outcome<br>Depression 1.50 (1.17-1.93)
Mendes de Leon et al (1996-USA) [53]	79.65.2	Female (73%)	A Prospective Study	1,103	ADL-related self-efficacy (FES) depression (CES-D)	Social support (two items about emotional support and instrumental support)	functional status (Katz ADL)	Mean (SD) of Baseline ADL<br>Low: 5.41 (0.9), Middle: 5.87 (0.4), High: 5.98 (0.1)	Adjusted model<br>Regression coefficient (SE)<br>ADL-related self-efficacy:<br>0.180 (.057), P<0.01<br>Baseline physical Performance<br>0.051(.007). p<.001<br>ADL (baseline)<br>0.593 (.048), p<.001<br>Emotional support<br>Not available, 0.092 (.082)<br>No need, -0.002 (.061)
Moreira et al (2016-Brazil) [54]	67–92	Men: 43 (41/7)<br>Women 60 (58.2)	A longitudinal study	103	Depression		functional capacity<br>(Katz ADL and IADL)	**ADL(2008)**<br>Mean (±SD)<br>5.73 (0.82)<br>**ADL(2010)**<br>5.52 (0.89)<br>p-value<br>0.0001<br>**IADL(2008)**<br>22.74 (2.68)<br>**IADL(2010)**<br>22.10 (3.46)<br>p-value<br>0.002	Multiple for IADL decline<br>Depression<br>OR (95% CI)<br>0.20 (0.04–1.03)

First author (year of publication-country)	**Age**	**Gender**	**Study design**	**Sample size**	**Determinants**		**Outcome**	**Descriptive measures**	**Analytic measures/conclusion**
					**Psychological**	**Social**			
								Subjects classified as low weight in 2008 (total, 7; IADL, 40% vs. ADL,6.7%) showed in 2010 a more prevalent IADL compared to ADL).<br>It was also verified for overweight older adults IADL 34.2% vs.<br>ADL, 23.7%.<br>For IADL decline (Depression) n (%)<br>Yes: 7 (50%)<br>No: 7 (50%), p=0.03	
Norburn et al (1995-USA) [55]	> 65		Longitudinal study	3485		Self-Care<br>Social support	Functional status<br>(BADL)<br>(MADL)<br>(IADL)	-	**Multivariable analysis**<br>**(Model 5)**<br>**b (SE)**<br>(Social support: MADL): -.01 (.36)<br>IADL:0.80 (.20) ***p< .01***<br>BADL:0.54 (.31)<br>**(Model 6)**<br>MADL: 0.07 (.42)<br>IADL: .76 (.20), p< .01<br>BADL:0.53 (.32)<br>**(Model 5)**<br>Level of IADL Disability(Slight disability: 0.91 (.21)<br>Mild disability:<br>1.30 (.39), p< .01<br>Moderate disability:<br>1.27 (.33), p< .01<br>Severe disability: 0.32 (.49)<br>**Model 6**<br>(Slight disability: 1.09 (-25), p< .01<br>Mild disability: 1.42 (.42), p< .01<br>Moderate disability:1.23 (.34) p< .01<br>Severe disability:0.37 (.48)<br>Level of BADL Disability<br>No disability (referent category)<br>**Model 5**<br>Slight disability: 0.59 (.19). p< .01<br>Mild disability:

First author (year of publication-country)	Age	Gender	Study design	Sample size	Determinants		Outcome	Descriptive measures	Analytic measures/conclusion
					Psychological	Social			
									1.55 (-62), p< .05<br>Moderate disability:0 .79 (.49)<br>Severe disability:0 .26 (.82)<br>**Model 6**<br>Slight disability: 0.85 (.20), p< .01<br>Mild disability: 1.59(.65)<br>Moderate disability: 1.16(.54)<br>p< .05<br>Severe disability:<br>.45(1.10)<br>Level of MADL Disability<br>No disability (referent category)<br>**Model 5**<br>Slight disability: 1.55 (.34), p< .01<br>Mild disability: 1.35 (.41), p< .01<br>Moderate disability:0 .88 (.45), p< .05<br>Severe disability:0 .11 (.65)<br>**Model 6**<br>Slight disability: 1.57 (.33), p< .01<br>Mild disability: 1.76 (.51), p< .01<br>Moderate disability:0 .82 (.49)<br>Severe disability: 1.19 (.87)
Patino et al (2021-USA) [56]	> 65	Female: 849 (54.9)<br>Male: 696 (45.1)	longitudinal cohort study	1545	Depressive symptoms		functional dependency	SPPBscore<br>With depressive symptoms<br>mean ± SD<br>8.6 ± 1.4<br>without<br>depressive symptoms<br>9.1 ± 1.4	
Pek et al (2020-Singapore) [57]	mean age 67.22 years	Female: 167(72)<br>Male: 62(27)	prospective cohort study	229		Social Frailty	functional status (BADL) (IADL)	functional status (BADL and IADL had respective median scores of 100 and 23, corresponding to the maximum score	BADL ^, max 100<br>BADL in social Non-Frailty, social Pre-Frailty, social Frailty were 100 (100-100), 100 (95-100), and 95 (92.5-100), respectively.<br>P=0. 004<br>SPF significantly associated with poor physical performance measured by SPPB adjusted OR (95% CI)<br>7.66 (1.43–41.14)<br>low physical activity<br>3.66 (1.67–8.02)

First author (year of publication-country)	Age	Gender	Study design	Sample size	**Determinants**		**Outcome**	**Descriptive measures**	**Analytic measures/conclusion**
					**Psychological**	**Social**			
									poor physical performance<br>17.51(2.63–116.58)<br>low physical activity<br>4.46(1.37–14.54)
Russo et al (2007- Italy) [58]	> 80	Female: 244(67)<br>Male: 120(33)	prospective cohort study	364	Depression		functional status		Adjusted means of physical function measures(dependent variable) according to depression:<br>(IADL scale score<br>No depression 2.84 (0.14) vs.<br>depression 3.72 (0.25) p=0.003<br>**(ADL scale score)**<br>No depression: 1.35 (0.13)<br>Depression: 1.75 (0.24)<br>*P*-value : .01<br>Participants with depressed mood presented a higher number of impaired IADLs (3.69; SE, 0.25) compared with participants with less than 3 depressive symptoms (2.85; SE, 0.14; P = .005)
SanchezMartinez et al (2016-Spain) [59]	≥ 65	Female: 309(50.9)<br>Male: 298(49.0 9)	longitudinal study ''Pen˜agrande cohort	607	Depression		Functional status (FS) (ADL)	Some 43.3% (95% CI: 39.3–47.4) of the study population experienced FS decline.<br>Physical functional status (At baseline,<br>n(%)(95% CI)<br>69.1% (65.4–73.0) had no disability,<br>15.2% (12.2–18.1) had mobility disability<br>and 15.7% (12.7–18.6) had ADL disability	Depression at baseline were associated with FS decline OR (95% CI): 2.92 (1.71–5.02).
Shankar et al (2017-London) [60]	> 60	over half were women	Longitudinal Study	3070		social isolation, loneliness	Functional Status	Analytic sample<br>Problems with 1 or more ADLS (%):<br>Baseline: 15.5%,<br>Follow-up: 18.8%	A unit increase in loneliness score was associated with a 1.08 times increase in the incidence rate of number of ADLs.<br>In fully and mutually **adjusted models,**<br>social isolation<br>IRR (95% CI): 0.96 (.88–1.02)<br>And loneliness: 1.06 (.52–2.15)<br>were found to be associated with a decrease in gait speed at follow-up:<br>Depression<br>1.44 (1.13–1.85)

First author (year of publication-country)	Age	Gender	Study design	Sample size	Determinants		Outcome	Descriptive measures	Analytic measures/conclusion
					Psychological	Social			
									Baseline difficulties with ADLs<br>1.71 (1.54–1.88)
Storeng et al (2018-Norway) [61]	60–69	Women<br>2738<br>(54.2)<br>Men<br>2312<br>(45.8)	Prospective cohort study	5050	Depression	Social participation	ADL/<br>IADL status	More participants reported needing assistance from another person in any of the IADLs (19.9%)<br>ADLs (2.4%).<br>A total of 20.3% needed assistance in any of the ADL/IADLs.	Depression was a risk factor for needing assistance in one or more basic ADLs<br>Adjusted OR(95% CI)<br>1.58 (0.91, 2.73)
Wang et al (2020-China) [62]	≥ 60	-	Longitudinal Study	4994	Depressive Symptoms		Functional status	Functional status (IADL)<br>N (%)<br>Worse<br>1108 (22.2)<br>No change<br>3031 (60.7)<br>Better<br>855 (17.1)<br>P-value<br><0.001	Adjusted OR (95% CI)<br>Change in cognitive function<br>(No change vs. better): 0.903 (0.689, 1.184)<br>Worse vs. better: 1.446 (1.279, 1.634)<br>Worsening cognitive function<br>(yes vs. no):<br>1.467 (1.305, 1.650)
Almeida et al (2017- Australia) [63]	70-87	1148 men	Prospective longitudinal cohort	1148	Depression	-	functional capacity<br>(BADL<br>IADL)	-	Adjusted OR (95% CI)<br>Men with than without history of past depression and impaired IADL as outcome: 58% (15%, 116%)<br>Current depression at the start of follow-up was associated with increased risk of impaired ADL<br>1.70 (1.23, 2.34)<br>IADL at the follow-up assessment<br>3.89(1.88, 8.05)<br>The associations between depression and sit-to-stand and step tests remained statistically significant (P=.001 and P = .006, respectively), as did the associations with at least 1 impaired ADL: 1.62(1.24, 2.12)<br>IADL: 2.36(1.53, 3.65)
Béland et al (1999-Canada) [64]	>65	men:<br>518<br>women:<br>766	Longitudinal	1284<br>1273 (Follow-up)	Depressive symptoms		Functional status	Prevalence of disability based on dependency in any of seven ADL items was 15.5%.<br>50% of the respondents were disabled in at least one of 10 IADLs	OR (95% CI)<br>Depressive symptoms (≥16 vs. <16)<br>IADL: 1.7(1.0–2.8)<br>ADL: 2.2(1.4–5.5)<br>Deceased: 2.3(1.2–4.3)<br>Depressive symptoms (No data vs <16:<br>ADL: 12 (1.5-100)

First author (year of publication-country)	**Age**	**Gender**	**Study design**	**Sample size**	**Determinants**		**Outcome**	**Descriptive measures**	**Analytic measures/conclusion**
					**Psychological**	**Social**			
									Deceased: 25.0(: 3.2-200)<br>P<0.05 for all.
Guo et al (2021- China) [65]	>50	men: 2694(52.2)<br><br>women: 2460(47.7)	Longitudinal	5154	-	Social isolation loneliness	functional disability (ADL and IADL)	New-onset ADL disability at follow-up, 861 (16.7%), New-onset IADL disability at follow-up, 990 (19.2%)	
Hajek et al (2017- Germany) [66]	>75	Follow up (wave 2: men 807(34.3) women 1542(65.6) Follow up wave 4 men<br><br>495(33.3) Women 989(66.6)	prospective cohort	Follow up wave 2: 2349 Follow up wave 4: 1484	-	Social support emotional support practical support social integration)	functional impairment (FI)	functional impairment (FI): wave 2: 4.7 ± 0.8, wave 4: 4.3 ± 1.2	**FI** decreased with increasing **social support** in the total sample<br>β = 0.05, P< 0.001<br>The effect on FI was most pronounced with the dimension **social integration**<br>β = 0.135 P= 0.000 whereas changes in practical support only affected FI in the total sample<br>β = 0.095, P= 0.014<br>and changes in **emotional support** only affected FI in men<br>β = 0.078, P= 0.047
Iwasa et al (2009- Japan) [67]	≥65	men 283(39.8) women 427(60.1)	prospective cohort	710	Depression	-	functional decline (BADL)	functional decline:<br>BADL: 306 (43.0%)<br>Higher-level competence: 516 (72.6%)	RR (95% CI)<br>Depression status and BADL decline<br>1.46 (1.13, 1.89)<br>and competence decline<br>1.56 (1.18, 2.04)
